# Portable Accessible MRI in Dementia Research: Ethical Considerations About Research Representation and Dementia-Friendly Technology

**DOI:** 10.1017/jme.2024.157

**Published:** 2024

**Authors:** Eran Klein, S. Duke Han, Paul Tuite, W. Taylor Kimberly, Mohit Agarwal

**Affiliations:** 1: OREGON HEALTH AND SCIENCE UNIVERSITY, PORTLAND, OR, USA; 2: UNIVERSITY OF SOUTHERN CALIFORNIA, LOS ANGELES, CA, USA; 3: UNIVERSITY OF MINNESOTA, MINNEAPOLIS, MN, USA; 4: MASSACHUSETTS GENERAL HOSPITAL, BOSTON, MA, USA; 5: HARVARD MEDICAL SCHOOL, BOSTON, MA, USA; 6: MEDICAL COLLEGE OF WISCONSIN, MILWAUKEE, MILWAUKEE, WI, USA

**Keywords:** Dementia, Alzheimer’s Disease, MRI, Underrepresentation, Disability

## Abstract

The introduction of portable MRI (pMRI) has the potential to directly impact dementia research and ultimately clinical care. In this paper, we explore two ethical challenges facing the introduction of pMRI in dementia research. The first is the need to ensure that pMRI enhances rather than undermines efforts aimed at improving ethnoracial representation in dementia research. The second is the need to implement pMRI in dementia research in a dementia-friendly way that attends to the social context and lived experience of people with dementia.

## Introduction

Portable magnetic resonance imaging (pMRI) is an emerging new technology.^1^ In contrast to standard MRI scanners which are typically massive and require both large energy supplies to maintain low temperatures and electromagnetic shielding and soundproofing, portable MRIs are compact, require less power and cooling, and are mobile.^2^ Portable MRI can take different forms or approaches. Low-field (0.01–0.1T) or ultra-low-field (<0.01T) MRI uses low-strength magnets and applies advanced data analysis techniques, while mid-field (0.01–1T) MRI uses smaller-than-standard magnets that produce non-uniform magnetic fields out of which images can be reconstructed.^3^ These new MRI technologies offer a kind of portability unimaginable with conventional MRI.

One area of research in which pMRI promises to have a particularly important role is in understanding and developing treatments for dementia. The US Department of Health and Human Services began implementing the National Plan to Address Alzheimer’s Disease in 2012. One of the six goals of the National Plan is “identifying effective treatments and preventative interventions for Alzheimer’s disease and related dementias (ADRD)”.^4^ As part of the Plan, National Institutes of Health (NIH) spending on ADRD research increased nearly 4.5-fold from FY 2015 ($631 million) to FY 2020 ($2.87 billion).^5^ Structural MRI has played an important role in ADRD research, including in the development of emerging monoclonal therapies in Alzheimer’s disease (AD).^6^ The development of pMRI offers new opportunities for conducting dementia research.^7^ The introduction of pMRI will usher in changes to where research can be conducted (e.g., parking lots, nursing homes, community centers), who can conduct research (e.g., small university researchers, companies, citizen scientists), and who can be research participants (e.g., people living in remote areas, underserved communities). While much work still needs to be done — such as correlating novel pMRI with decades of data derived from higher-field MRI (e.g., 1.5T and 3T) — the development of pMRI has the potential to significantly change the current practice of dementia research.

The emergence of pMRI raises new questions about ethical implementation of this new technology, including competent MRI operation and research design, oversight, safety, diversity of research participants, artificial intelligence, science communication, privacy, access, and ownership of pMRI data.^8^ In the context of dementia research, addressing these kinds of ethical questions and others^9^ is made all the more complicated by two contextual features of dementia research and imaging: a history of underrepresentation in dementia research, particularly dementia neuroimaging research, and the “unfriendliness” of MRI in research and clinical care experienced by people living with dementia.

In this paper, we explore how pMRI intersects with problems of underrepresentation in dementia research and experiential obstacles to access and tolerability of MRI in people living with dementia. We argue that the ethical implementation of pMRI in dementia research will depend on attending to these challenges and developing appropriate strategies as this technology evolves.

## I. Anticipating the Role of pMRI in Dementia Research

Dysfunction in different domains of cognitive function (memory, language, executive function) can present as varying changes in behavior or cognitive performance that affect daily life.^10^ By recent estimates, approximately 55 million people worldwide live with dementia and the prevalence is increasing internationally.^11^ There are different causes of dementia with the most common being Alzheimer’s disease. The principal risk factor for Alzheimer’s disease is age, with 3% of individuals over 65 years old having AD, 17% of people over 75 years old, and 32% of individuals over 85 years old.^12^ In a constantly changing and ever evolving landscape regarding the pathophysiology of Alzheimer’s disease and other types of dementias such as Lewy body dementia, frontotemporal degeneration, and vascular dementia, the exact causes of why and how dementia develops in different individuals remains an intense area of research.

… [A] more compelling reason to anticipate the use of pMRI in ADRD research relates to the potential for pMRI to shift *who* participates in ADRD research and *how* they participate. Specifically, pMRI has the potential to make research participation more accessible to populations currently underrepresented in dementia research and lessen some of the burdens experienced by people living with dementia when getting an MRI.

Structural MRI has been a standard part of dementia research for decades. This role has evolved in concert with the evolution of both neuroimaging technology and the pathophysiologic understanding of dementia, hereafter referred to as Alzheimer’s disease and related dementias (ADRD).^13^ Structural MRI has been the preferred imaging modality in research (vs. computed tomography (CT)) for both technical and safety reasons.^14^ In neuroimaging, MRI has the unique advantage of providing excellent anatomic detail and demonstrating pathology through its ability to characterize tissue by the use of different pulse sequences. High resolution 3D anatomic data provided by MRI can be immensely helpful in determining areas of brain volume loss (atrophy), which is the end result of neurodegeneration seen in the disease processes that clinically manifest as dementia. These inherent advantages of MRI trump the use of CT in much ADRD research. Additionally, MRI is readily available at most research institutions. Three principal roles of structural MRI in ADRD research are (1) screening for inclusion/exclusion criteria of research participants; (2) evaluating outcome measures (e.g., brain atrophy); and (3) monitoring for adverse events. In this section, we anticipate ways that pMRI can contribute to screening, evaluating outcome measures, and adverse event monitoring in ADRD research.

Portable MRI has potential use as a screening tool in ADRD clinical research. Most clinical research in ADRD requires excluding confounding conditions.^15^ Coupled with other data, like clinical syndrome and laboratory tests, neuroimaging is helpful for identifying ADRD mimics, like brain tumors, normal pressure hydrocephalus, central nervous system infections, inflammatory conditions, traumatic brain injury, and metabolic derangements (e.g., vitamin deficiencies such as thiamine). Structural brain changes, particularly the patterns of regional brain atrophy, can sometimes help distinguish between AD, dementia with Lewy bodies, frontotemporal disease, and other causes. Structural MRI has played a central role in clinical ADRD research by excluding confounders of cognitive and behavioral change in potential study enrollees.

Portable MRI also has the potential to be used for evaluating whether research participants meet inclusion criteria. Most clinical research in ADRD requires a diagnosis (or absence of a diagnosis) of ADRD. Establishing an accurate diagnosis in ADRD research has been critically important and also challenging. Measuring effects of interventions in ADRD is complicated because much clinical research outside of dementia research (say, many oncology trials) works on timelines of weeks to months. But clinical changes in ADRD can be slow and take place over years. A recent systematic review found the mean survival time from AD symptom onset to be 7.6 years, and 5.8 years from diagnosis.^16^ Because of the relatively slow trajectory of AD, demonstrating clear benefit during a clinical trial has been challenging. Other forms of ADRD, such as dementia with Lewy bodies or progressive supranuclear palsy, have a steeper clinical trajectory, but still progress with a longer timeline than many non-neurodegenerative conditions. Enrolling large numbers of participants or running studies for extended periods of time (e.g., multiple years) has not been considered feasible.^17^ The ability to more accurately sort potential study participants by etiology of dementia may allow for design of studies that enroll fewer, more narrowly diagnosed participants. This underlines the importance of highly accurate diagnosis at enrollment.

This has led the ADRD research community to undertake efforts to develop strict diagnostic criteria for ADRD which have evolved over time.^18^ The role of neuroimaging as part of these criteria has similarly evolved. For instance, in moving Alzheimer’s disease research to a biological definition of AD, neuroimaging is recommended as one of the biomarkers that can serve as a proxy for the neuropathology of AD (along with amyloid deposition and neurofibrillary tau tangles).^19^ While MRI findings from fixed MRI, such as degrees of regional atrophy, have not traditionally been used as inclusion criteria for ADRD research, a shift to biomarker-based research, including neuroimaging biomarkers from pMRI, will open new opportunities for shaping inclusion criteria of ADRD studies.

Portable MRI has the potential to measure outcomes of interest in ADRD research, such as atrophy of brain structures. Progressive cerebral atrophy, especially of the medial temporal lobe structures and in particular the hippocampi, is a characteristic feature of Alzheimer’s disease, often present even before symptoms. Evidence of atrophy seen on MRI tracks both cognitive impairment^20^ and the pattern of progressive neurofibrillary tangle pathology seen at autopsy, starting in the entorhinal cortex and hippocampus, spreading to the basal temporal lobe and paralimbic cortical areas, and then continuing into association cortices.^21^ Because of the structural correlations of atrophy with the density of neurofibrillary tangles and therefore neuropathology, MRI has been widely used in ADRD research.^22^ While cognitive, functional and other biomarker outcomes have been the predominant outcome measures in ADRD research, MRI also has been used to evaluate drug effects on neurodegeneration (e.g., hippocampal loss).^23^

The third role that pMRI may play in ADRD research is safety monitoring. Similar to its use in screening for study entry, MRI has been used to evaluate unexpected events during research studies (e.g., cerebrovascular events, central nervous system infections, tumors). The safety monitoring role of structural MRI has been particularly prominent in the development of immunomodulatory therapies, such as aducanumab, lecanemab, and donanemab. These new therapies have been associated with episodes of brain edema or hemorrhage (i.e., amyloid-related imaging abnormalities (ARIA)).^24^ Well-developed research protocols for monitoring the development of symptomatic and asymptomatic ARIA have been part of clinical trials.^25^ These imaging findings have been a critical consideration in the research design and in the regulatory approval of these drugs. In addition, concerns about efficacy and safety related to ARIA led Centers for Medicare and Medicaid (CMS) to restrict coverage of aducanumab to patients concomitantly enrolled in a clinical trial.^26^ Coverage for lecanemab required enrollment in a registry that documents adverse events, including ARIA.^27^

So why give special consideration to the role of pMRI when fixed MRI is already in wide use in ADRD research and will for the foreseeable future produce a wider range of sequences and higher quality of images? There are several reasons for this. Some are technical. Not all Alzheimer’s disease research questions will require the capabilities of fixed MRI. For example, depending on the particular field strength and future technological developments, some pMRI may be sufficient for evaluating inclusion and exclusion criteria in ADRD studies (e.g., excluding structural lesions, such as stroke or hemorrhage). Secondly, it is possible that some forms of pMRI may have the capability to detect ARIA or other structural or functional biomarkers related to ARIA (e.g., atrophy) and thereby facilitate the extensive and challenging task of monitoring for disease-related complications. While the necessary studies to compare pMRI to fixed MRI sensitivities to detect ARIA or associated structural biomarkers have not yet been performed, they presumably will happen.^28^ But a more compelling reason to anticipate the use of pMRI in ADRD research relates to the potential for pMRI to shift *who* participates in ADRD research and *how* they participate. Specifically, pMRI has the potential to make research participation more accessible to populations currently underrepresented in dementia research and lessen some of the burdens experienced by people living with dementia when getting an MRI.

## II. Representation and MRI in Dementia Research

The introduction of pMRI in ADRD research will not happen *de novo* but against a backdrop of the history of ADRD research as well as social and scientific understandings of dementia more generally. An exploration of the potential of pMRI to affect — maybe even transform — the practice of ADRD research is best done by contextualizing it within this history. An important aspect of this history is underrepresentation of some groups relative to others within ADRD research.

The risk for developing dementia is not evenly distributed across populations. For instance, it is increasingly recognized that some groups have an increased risk of developing dementia.^29^ By one estimate, 18.6% of Black Americans, 14% of Hispanics, and 10% of Whites over age 65 are diagnosed with Alzheimer’s disease.^30^ These differences are poorly reflected in the clinical trial populations in ADRD research. For instance, Black Americans constitute only 2% of Alzheimer’s disease clinical trial participants.^31^ Poor representation of minoritized communities is mirrored in neuroimaging research related to Alzheimer’s disease.^32^ The lack of diverse representation in ADRD research across lines of ethnicity, race, economic status, and geographic location is increasingly recognized. For instance, the NIH Revitalization Act of 1993 established guidelines for inclusion of women and individuals from minority races and ethnicities^33^ and the National Alzheimer’s Project Act formally committed to increasing enrollment of underrepresented groups.^34^ Large dementia cohort studies have made efforts to improve recruitment of participants from minority groups.^35^ Despite these efforts, ADRD trials have struggled to demonstrate improvement in representation.^36^ For example, Black Americans are underrepresented within the critical studies on new Alzheimer’s disease therapies.^37^

There are various contributors to underrepresentation in clinical research.^38^ In ADRD research, these include differences in recruitment and retention,^39^ but also include systematic exclusion based on ADRD clinical trial eligibility criteria. For instance, a systematic review of international ADRD trials found that the reduced ethnodiversity of trials was in part due to exclusion of people with psychiatric conditions and cardiovascular disease or those without available caregivers (because studies often require willingness of caregivers to assist with trial participation), all factors that vary across ethnic and racial groups.^40^

Underrepresentation in ADRD research has important implications. Underrepresentation can lead to a lack of knowledge production about efficacy and perpetuation of group and individual harms^41^ and this is particularly true in ADRD research given existing racial/ethnic disparities in Alzheimer’s disease diagnosis and treatment.^42^ There is risk of perpetuating harms even as promising new therapies for Alzheimer’s disease emerge. For instance, given known risks of brain hemorrhage from monoclonal antibody therapy, people with comorbidities associated with bleeding were excluded from pivotal trials. While such exclusions have a reasonable safety rationale, when contextualized against a background of differential prevalence of dementia and these comorbidities, such exclusion may further widen gaps in understanding dementia and future care. As noted by published appropriate- use recommendations, “Excluding patients with some comorbidities and treatments including anticoagulants, severe cerebrovascular disease, and others may result in disproportionately excluding patients from underrepresented groups including Black/African Americans, Latinos, Asians, Native Americans and Pacific Islanders, and others with adverse determinants of health from being considered as candidates for lecanemab therapy”.^43^

## III. Access and Tolerability of MRI in People Living With Dementia

Logistical access to and the ability to tolerate undergoing MRI imaging related to diagnosis and treatment are challenges frequently faced by people living with dementia and their family members. Similar challenges confront people living with dementia who wish to participate in dementia-related research.

### A. Access to MRI

Participation in ADRD clinical research typically requires the ability to travel to the site of a fixed MRI machine. Traditionally, fixed MRI used in research has been located at academic medical centers and large health care systems. Travel to facilities with fixed MRI can be difficult for people with dementia. Driving is a privilege granted and regulated by municipalities. Medical conditions that can impair driving ability, like dementia, lead to restrictions or revocation of driving privileges. Most drivers with a diagnosis of dementia eventually lose both the ability and legal authorization to drive. Individuals with dementia who do not drive may have difficulty navigating or lack confidence to use public transport on their own. Dementia makes travel to a fixed MRI for research participation an additional hurdle. Though family members or others can provide transportation to people living with dementia if they wish to participate in a clinical trial, this can be a burden, particularly if doing so requires taking off work or deferring other responsibilities. Adult-child caregivers of people living with dementia are more likely to still be working.^44^ For those living in institutional settings, such as assisted living or memory care, assigning someone to take a person living with dementia to get an MRI may divert caregiver or other resources away from others who are in need.

Travel itself is also difficult for people living with dementia. People with dementia often become reliant on routine. Changes to sleep, eating, or other daily schedules can be particularly difficult for people living with dementia, and lead to mood or cognitive side effects, such as agitation or depressive symptoms. These psychological effects are disruptive and emotionally difficult, not just for the person living with dementia but for family members or other caregivers who accompany them to get an MRI. Seeing one’s loved one put under stress can be a particularly wrenching experience. During a trip from home to a fixed MRI, a person living with dementia can forget where or why they are traveling and repeatedly ask questions about this, leading to stress on the part of a person living with dementia and the person accompanying them. People living with dementia are typically older and can have co-morbidities that affect mobility, such as parkinsonism or cerebrovascular disease. As such, getting in and out of vehicles can be hard if individuals have bradykinesia, paresis, chronic pain, or apraxia. The difficulties of traveling to an MRI can be exacerbated by socioeconomic factors and geography (e.g., greater distance to the MRI may mean that a person living with dementia and their transportation partner have to dedicate more time to participate in a clinical trial involving an MRI).^45^

Access to MRI involves not just getting to a facility with an MRI but traveling to the MRI after arriving at the facility. Getting around MRI facilities can be difficult for a person living with dementia. As Manietta notes, “hospitals are in general an unbefitting environment for people with dementia because of functional care, processes, architecture, noise and the presence of strangers.”^46^ Navigating large, busy environments can be difficult for people who struggle with processing information, remembering directions, or visuospatial skills. Signage may be limited or confusing, leading people living with dementia to get lost on the way to radiology departments.^47^ And even once arriving at a radiology department, obstacles may remain. Research suggests that people living with dementia have bad experiences with radiology.^48^

Portable MRI has the potential to help address underrepresentation in dementia research by making MRI more accessible. pMRI may make it easier for groups underrepresented in dementia research to participate in research by reducing the burdens on people living with dementia and support partners currently associated with traveling to and getting around facilities with fixed MRIs. Even in individuals with mild cognitive impairment (often a precursor of dementia) or mild dementia in which driving ability is minimally affected, anxiety about driving can be high and lead to lack of driving confidence, and in turn a reluctance to enroll in research that requires significant travel. By providing a more conveniently located MRI, pMRI provides the potential for greater research participation. It is possible that the convenience of pMRI may enhance enrollment in future dementia therapy trials. As described previously, MRI plays a critical role in assessing, sometimes emergently, potential side effects (e.g., ARIA) of therapies. If pMRI dramatically reduces the burden on research participants and family members to monitor potential side effects, this may alter the cost-benefit considerations for those contemplating study enrollment.

Even in individuals with mild cognitive impairment (often a precursor of dementia) or mild dementia in which driving ability is minimally affected, anxiety about driving can be high and lead to lack of driving confidence, and in turn a reluctance to enroll in research that requires significant travel. By providing a more conveniently located MRI, pMRI provides the potential for greater research participation.

It is worth acknowledging that the promise of greater accessibility of pMRI will not be achieved through mere improvement of physical proximity. For pMRI to achieve greater accessibility than fixed MRI, it will have to deliver on all of the five key dimensions of medical device accessibility elucidated by the World Health Organization: geographical, temporal, financial, cultural, and digital.^49^ As Savold notes, accessibility with respect to ADRD research requires that the places where research is conducted be trusted by the community.^50^ This highlights the need to attend to histories of trust and mistrust when researchers physically bring pMRI into new environments.

### B. Tolerability of MRI

The difficulty of undergoing an MRI can be significantly exacerbated in people with dementia. An MRI scan conducted for research can last 15–30 minutes, sometimes longer. Individuals are often required to lie still and have their head tightly secured in the machine. The machine is loud and typically requires wearing protective hearing devices. For people with dementia who experience confusion and anxiety, this process of undergoing an MRI can be particularly difficult. The inability of a person living with dementia to tolerate MRI has been shown to have significant financial costs.^51^ If the difficulty of tolerating an MRI is taken as a reason to exclude someone from a study or if participants choose not to participate out of fear of these MRI-related experiences, this affects who is represented in ADRD research.

MRI causes anxiety and claustrophobia in people with and without dementia. MRI is contraindicated in people with claustrophobia and as such is an exclusion for research studies requiring an MRI. There are interventions that sometimes can be used for claustrophobia and anxiety associated with MRI, but these may not work or may have adverse effects on people living with dementia. For instance, anxiolytics (e.g., lorazepam, valium) may cause paradoxical reactions in people living with dementia, such as delirium or agitation.^52^ This is unfortunate since generalized anxiety is often comorbid with dementia; individuals who most need an effective anxiolytic may be least likely to have an effective one available to them.

As previously noted, people living with dementia are at increased risk of confusion and disorientation when traveling long distances to obtain an MRI, but they are also at risk of confusion due to the process of getting an MRI itself. Activities that some people use to cope with MRI, such as watching a video or listening to music, can be disorienting for people with dementia. And even falling asleep during the MRI, which is often a “bonus” for people without cognitive impairment, can be difficult for people living with dementia who awake disoriented. Moreover, the loud noise that is part of getting an MRI can be disorienting in itself for people living with dementia. MRI in conscious individuals requires some level of participation and following of directions (e.g., limiting one’s movement). People living with dementia who have receptive or expressive aphasia may have difficulty understanding directions or expressing their needs (e.g., to report discomfort, to ask to pause a scan), and even those without language impairment but who have amnestic symptoms can forget the purpose of the study or how long it will last while in the middle of it. This can lead to frustration or miscommunication with radiology staff.^53^ While in day-to-day life, carers and care partners can often reduce episodes of confusion and anxiety associated with dementia through reassurance, reorientation, or just presence, this is not possible with fixed MRI, which precludes others from being in the immediate vicinity of a high-field MRI machine. Research with patients, families, clinicians and MRI departments demonstrates that MRI is, in many ways, not “dementia-friendly”.^54^

## IV. Realizing the Potential of pMRI in Dementia Research: Research Representation and Dementia-Friendly Imaging

Portable MRI has the potential to change who participates in dementia research and improve the experience of those who participate. Both are ethical imperatives for the development of pMRI. Portable MRI offers significant advantages over fixed MRI with respect to location of scanning and ease of scanning. As a result, pMRI may allow individuals who do not currently participate in ADRD research to do so and to alleviate significant psychological or other burdens experienced by people living with dementia who do participate in research involving MRI.

Portable MRI has the potential to improve scientific understanding of ADRD in previously underrepresented communities, and in turn reduce disparities in diagnosis and care. For instance, if individuals have greater access to and better experiences with MRI through participating in ADRD research, this may lower the bar to accessing MRI for clinical diagnosis and care in the future. An analogous example can be found in the field of multiple sclerosis (MS). The use of immunomodulatory infusions in MS research began in the 1990s and accelerated in the decades after.^55^ Infusions were a common part of MS research and as immunomodulatory drugs were approved for clinical use, the MS community of patients, families, and providers had become acculturated to going to infusion centers for periodic treatment, sometimes preferring this to more frequent oral or injectable therapies.^56^ If pMRI becomes a common method in ADRD research to monitor effectiveness or side effects of new dementia interventions (e.g., immunomodulatory drugs) in underrepresented populations, individuals in these groups also may become more comfortable pursuing clinical interventions that require MRI diagnosis or treatment monitoring in the future. There is no guarantee of this. As has recently been argued, community involvement in how pMRI is developed and deployed will be critical to ensuring uptake of pMRI in research and to ensuring that the downstream benefits are equitably shared with members of all communities.^57^

Portable MRI also has the potential to help shift neuroimaging research toward more dementia-friendly practices. Dementia and dementia-related disability have traditionally been viewed through a medical lens. Through this lens, dementia in its various forms is taken to cause cognitive and behavioral impairments, which in turn result in disabilities (e.g., inability to work, drive, manage finances, make autonomous decisions about research participation, etc.). Disability theorists and others have challenged this medical framework and offer an alternative social model of dementia and disability.^58^ In contrast to a medical model, a social model of disability in the context of dementia understands that “both the health condition itself, and the social responses to it, generate the disability.”^59^ This social model of dementia disability in part underwrites efforts to make clinical care in dementia more “dementia-friendly”.^60^ Dementia-friendly has been defined as “the creation of supportive, inclusive, and enabling environments that maximize independence through collaboration with diverse community stakeholders.”^61^ Applications of a dementia-friendly approach include designing dementia-friendly neighborhoods, nursing homes, and health care facilities, among others.^62^

The need and value of applying a dementia-friendly and person-oriented approach to the conduct of dementia research is beginning to be recognized.^63^ What has not yet been explored is the possibility that pMRI can play a role in making research more dementia-friendly. As has been suggested, pMRI may alleviate some of the harms related to anxiety and confusion when a person living with dementia gets an MRI.^64^ The very nature of a pMRI is that it reduces the risks for subjects undergoing scanning and anyone in close proximity. For instance, a research participant with dementia who is undergoing a pMRI can hold the hand of a loved one or communicate because of reduced noise levels or be able to fix their gaze on a family member’s face through an open aperture; this may be a significant comfort to someone with dementia undergoing a pMRI as well as for those who are accompanying them. If pMRI is or can be made more dementia-friendly, this may open new avenues of investigation of clinically important questions that are currently under-investigated using fixed MRI, like psychosis or physical aggression, or dementia-associated delirium.^65^ More broadly, pMRI as a dementia-friendly technology may facilitate a welcome shift in how the challenges facing people living with dementia who participate in research are understood: from the current paradigm of seeing obstacles to effective research participation of people living with dementia as located in the individual with dementia (e.g., in their “cognitive deficits” or “behavioral problems”) to recognition of the failure to design research practices and technologies that enable research participation of people living with dementia.

## Conclusion

The introduction of pMRI presents a valuable opportunity for the field of ADRD research. ADRD research has struggled to diversify representation despite expressed commitments to address this failing. Portable MRI offers the possibility of reconfiguring at least one of the traditional barriers to research participation (e.g., location). In addition, MRI has not traditionally been a dementia-friendly technology, but pMRI offers ways to significantly improve the experience of people living with dementia who get an MRI as part of dementia-related research. Portable MRI, its capabilities, and potential uses in dementia research are only starting to come into view. This is all the more reason to attend to the challenges facing people with dementia who wish to participate in research, and creatively imagine ways that technologies like pMRI may facilitate this participation.
